# Acute and Subchronic Oral Safety Profiles of the Sudarshana Suspension

**DOI:** 10.1155/2020/2891058

**Published:** 2020-11-28

**Authors:** Weerakoon Achchige Selvi Saroja Weerakoon, Pathirage Kamal Perera, Kamani Samarasinghe, Dulani Gunasekera, Thusharie Sugandhika Suresh

**Affiliations:** ^1^Department of Ayurveda Obstetrics and Paediatrics, Institute of Indigenous Medicine, University of Colombo, Colombo, Sri Lanka; ^2^Department of Ayurveda Pharmacology and Pharmaceutics, Institute of Indigenous Medicine, University of Colombo, Colombo, Sri Lanka; ^3^Department of Pathology, Faculty of Medical Sciences, University of Sri Jayewardenepura, Nugegoda, Sri Lanka; ^4^Department of Paediatrics, Faculty of Medical Sciences, University of Sri Jayewardenepura, Nugegoda, Sri Lanka; ^5^Department of Biochemistry, Faculty of Medical Sciences, University of Sri Jayewardenepura, Nugegoda, Sri Lanka

## Abstract

*Sudarshana* powder (SP) is an Ayurvedic preparation, which contains 53 herbal ingredients along with 50% of *Andrographis paniculata* and is clinically used with bees honey. This study was aimed to determine the safety profile of the SP, and its novel preparation Sudarshana suspension (SS) on male Wistar rats and tolerance studies were conducted for healthy adult volunteers. Acute and subacute toxicity studies of the SS and hot water extract of SP were assessed in Wistar rats by observing the general behavior, analyzing biochemical and haematological parameters, and pathological observation. Healthy consented adult volunteers (*n* = 35) of either sex were selected, and tolerance studies of SS were tested by measuring the biochemical and haematological parameters. There were no significant (*p* > 0.05) changes observed in the treated animals with SS and hot water extract of SP compared with control in body weights, food intake, and water consumption as well as the biochemical and haematological parameters. Histopathological studies revealed no significant (*p* > 0.05) changes in the liver, heart, and kidney tissues. The experimental results suggest that novel formulation SS was potentially safe for chronic administration in rats, and no significant differences (*p* > 0.05) were observed in tested parameters on day 3 and day 8 when compared to the day 0 (baseline) values in healthy volunteers. Healthy volunteers did not report any adverse effects or any other complications during the treatment period and the follow-up period. Therefore, it can be concluded that the novel preparation Sudarshana suspension does not cause any significant toxic effects on the blood parameters in animal and human models.

## 1. Introduction

Sudarshana powder (SP) is an effective antipyretic Ayurvedic preparation, widely used in Sri Lanka as well as in India from the very early beginning of Ayurveda treatment. Sudarshana powder (*Sudarshana churna*) is mentioned in the Ayurvedic Pharmacopeia complied under Sec. 41 (2) (c) of Ayurveda Act no 31 of 1961 by the Ayurvedic Pharmacopeia Committee under the direction of the Ayurvedic Research Committee. During the early beginning of Ayurveda treatment in Sri Lanka, the main ingredient of the SP was *Swertia chirata* which was later replaced by *Andrographis paniculata* (Burm. F.) Nees in Sri Lanka. Presently, the SP contains *Andrographis paniculata* (Burm. F.) Nees (50%) along with other 52 ingredients (50%) [1]. This powder is being widely used with bees honey in all Ayurvedic hospitals and dispensaries for adults as well as in pediatric patients including National Ayurveda Teaching Hospital, Borella, Sri Lanka. Bees honey is recommended as an *Anupana* (vehicle) for SP in the pediatrics age group in Ayurveda [2]. No reports on adverse effects have been reported of this mixer clinically.

It is recommended for all types of fever and common cold [3]. It is used traditionally as antimalarial, antiviral, and antipyretic formulation for which it is highly effective [1]. SP has been clinically used with bees honey to mask its bitter taste, but there is no ready-to-use product with the correct amount of bees honey in current dosage forms. Therefore, this powder was developed into user-friendly ready-to-use standard Ayurveda suspension using bees honey *viz* SS. SP and bees honey is the only ingredients of this novel preparation. There are no other added substances in this novel preparation. Suspension forms of drugs are an easy way to administer to the pediatrics and elderly who have difficulty in taking drugs in tablet or capsule forms. But, to date, there is no scientific research conducted for evaluating the safety of SP and SS. Thus, this study was aimed to determine the safety profile of the SP and SS effects after acute and chronic oral administration in male Wistar rats, and tolerance studies were conducted in healthy volunteers.

## 2. Materials and Methods

### 2.1. Collection of Materials and Preparation of Sudarshana Powder

All the ingredients were collected from the Ayurveda Drug Cooperation, Sri Lanka, and authentication of ingredients was done at the Institute of Indigenous Medicine, University of Colombo, Sri Lanka (Specimen no. 102). *Sudarshana* powder was prepared according to Ayurveda Pharmacopoeia [1, 4] at the pharmacy of the Institute of Indigenous Medicine, University of Colombo, Sri Lanka.

### 2.2. Preparation of Extracts

Aqueous extracts of SP were prepared by infusion with hot water, occasional shaking, and filtration (preparation method of Phanta Kasaya in Ayurveda medicine) [1].

### 2.3. Preparation of Suspension

*Sudarshana* powder is converted to *Sudarshana* suspension (SS) using bees honey, according to the Ayurvedic Mana *Paribhasha* (the method of syrup or suspension preparation) [5].

### 2.4. Animals

Healthy adult male Wistar rats (200–250 g) were used in the study. The animals were kept in plastic cages (two per cage) under standardized animal house conditions at the animal house, Faculty of Medical Sciences, University of Sri Jayewardenepura, Sri Lanka, with continuous access to standard pelleted feed and tap water.

### 2.5. Ethical Approval for Animal Studies

All experiments in rats were carried out in accordance with the recommendation of the guidelines for care and use of laboratory animals, and the project proposal was approved (no. 591/11) by the Ethics Review Committee of the Faculty of Medical Sciences, University of Sri Jayewardenepura, Sri Lanka (http://medical.sjp.ac.lk/index.php/ethics-review-committee-introduction).

### 2.6. Dosage and Administration of Drug to Animal Models

The doses of drugs corresponded to the normal therapeutic dose administered to adult humans as calculated, based on relative surface areas of humans and each individual animal. The dosage was calculated based on its weight, and then, it was multiplied by 6 (conversion factor, km: Factor for converting mg/kg dose to mg/m^2^ dose) [6].

### 2.7. Acute Oral Toxicity Study

Wistar rats were kept for a minimum of 5 days prior to oral administration at the animal house, Faculty of Medical Sciences, University of Sri Jayewardenepura, Sri Lanka, to allow for their acclimatization to the animal house conditions. The animal room was ventilated with a 12°h cycle of day and night light conditions, and the temperature was maintained at approximately 25°C. Tap water and food were readily accessible to the rats throughout the study; however, prior to the oral administration of the single doses of Sudarshana suspension and infusion of Sudarshana powder, all animals were subjected to a short fasting period of 5 h [7, 8].

The acute toxicity [9] of SS and SP was compared with that of the control group, distilled water given at a rate of 1 ml/rat. There were 6 rats in each group. Test group 1 received a single dose of SS (4 ml/kg), and Test group 2 received a single dose of SP (0.5 g/kg). Treated animals were deprived of food and water for 2 h to assess the general behavior of rats and thereafter during a period of 48 h for dead animals. During a 48 h period of observation, the body weight changes and food and water intakes were recorded followed by observation for 2 weeks for possible signs of toxicity and deaths and the latency of death. Blood was collected for biochemical and haematological analyses after 48 h and 14 days, and creatinine, alkaline phosphatase, alanine aminotransferase (ALT), aspartate aminotransferase (AST), gamma-glutamyl transferase (*γ*-GT), and haemoglobin (Hb) levels were analyzed by the spectrophotometer. BIOLABO kits from France were purchased from Analytical Instrument (Pvt) Ltd., Colombo.

### 2.8. Subchronic Oral Toxicity Study

The SS and SP were compared with the control in three groups of rats, 6 rats in each. The control group received distilled water as a vehicle, Test group 1 received a single dose of SS (4 ml/kg), and Test group 2 received hot water extraction of SP (0.5 g/kg), single dose daily, for 42 consecutive days.

The body weights and the food and water intakes were recorded weekly. After 42 days of administration, blood was collected for haematological and biochemical analyses. The haematological parameters (i.e., WBC, RBC, Hb, PCV, MCV, MCH, MCHC, RDW, MPV, and platelets) and the biochemical parameters including creatinine, alkaline phosphatase, alanine aminotransferase (ALT), aspartate aminotransferase (AST), gamma-glutamyl transferase (*γ*-GT), and urea were evaluated. Reagent BIOLABO kits from France were used for the analysis, and the animals were sacrificed to harvest the liver, heart, and kidneys and weighed individually. Macroscopic and microscopic analyses were carried out.

### 2.9. Statistical Analysis

Results are presented as mean ± standard error of mean (SEM). Student's *t* test was used for statistical comparison of data between groups. Differences were considered significant at *p* ≤ 0.05.

### 2.10. Ethical Clearance Process

The clinical study on healthy volunteers was approved by the Ethics Review Committee, Faculty of Medical Sciences, University of Sri Jayewardenepura, Sri Lanka (ref. no. 775/13), and Ethics Review Committee, Institute of Indigenous Medicine, University of Colombo (ref. no. ERC 12/11), Sri Lanka. A clinical study was registered at the Sri Lanka Clinical Trial Registry ((reg. no. SLCTR/2015/005) (http://slctr.lk/trials/304) and WHO International Clinical Trials Registry (http://apps.who.int/trialsearch/Trial2.aspx?TrialID=SLCTR/2015/005)).

### 2.11. Safety Effect of *Sudarshana* Suspension in Healthy Volunteers

Safety effect on hepatic and renal functions of the novel preparation Sudarshana suspension in healthy volunteers was tested by measuring the key hepatic enzymes alanine aminotransferase (ALT), aspartate aminotransferase (AST), gamma-glutamyl transferase (gamma GT), alkaline phosphatase, haemoglobin content (Hb), urea in serum, and creatinine.

### 2.12. Study Design

This study is an analytical interventional single-arm study where all volunteers were recruited until the required number was achieved.

### 2.13. Sample Size

Thirty-five healthy adult volunteers [10] were selected by an open advertisement.

#### 2.13.1. Inclusion Criteria

1. Age group of 18–60 years at the time of enrollment, of either sex with weight less than 60 kg
2. Those having no known systemic disorders, such as hypertension, diabetes mellitus, hypercholesterolemia, and chronic arthritis
3. Those having no history of drug allergy
4. Those having no history of intolerance to SP or similar compounds
5. Women should be neither pregnant and nor breast feeding
6. Only those who can write and read languages of English or Sinhala.

#### 2.13.2. Exclusion Criteria

1. Less than 18 years of age and over 60 years at the time of enrollment, of either sex
2. Weight more than 60 kg
3. Concurrent treatment with any Ayurveda or Western medicine

### 2.14. Determination of Appropriate Dose of the SS

The dosage of the test drug was determined by calculating the dose per adult equivalent to the normal paediatric dosage proportionate with the body weight of a child.

### 2.15. The Procedures and Duration

Written consents were obtained from the participants before initiating the study, and the information sheets and diary sheets were distributed to the participants in their own language.

Sudarshana suspension was prepared at the pharmacy of the Institute of Indigenous Medicine, University of Colombo, Sri Lanka, and filled in 500 ml air-tight, amber, labeled glass bottles.

Adult volunteers were given four doses of drug at six-hour intervals per day for 7 consecutive days. The dosage of drug was determined according to the body weight of the volunteer. 3 ml of blood was drawn on days 0, 3, 8. The liver and renal functions tests (ALT, ALP, AST, *γ*GT, creatinine, and urea) were done using the automated biochemical analyzer (Kone) at the Department of Biochemistry. Hb was analyzed by using the spectrophotometer at the Research Lab, Department of Biochemistry, FMS, USJ. BIOLABO kits were purchased from Analytical Instrument (Pvt) Ltd., Colombo. In each subject, basic epidemiological and anthropometric data (age, sex, and weight) were recorded. After the first and last doses, palatability was recorded using a modified five-point scale [11]:

  0 = severely dislikes the taste and vomits
  1 = dislikes the taste but does not vomit
  2 = takes the drug rather unhappily
  3 = takes the drug without any complaint
  4 = likes the taste a lot

## 3. Results

### 3.1. Acute Oral Toxicity

The results of the acute toxicity study showed no signs of toxicity such as general behavioral changes or mortality. No abnormalities were detected in any of the tested blood parameters as well as body weights, food intake, and water consumption in the SS and SP groups when compared with the control group. The effects of SS and hot water extraction of SP after 48 hours of oral administration are summarized in Table 1.

The effects of SS and hot water extraction of SP in biochemical parameters after 14 days of oral administration are summarized in Table 2.

### 3.2. Subchronic Oral Toxicity

#### 3.2.1. Effect of the Oral Administration of SP and SS on the General Behavior of the Rats

**Figure 1:**
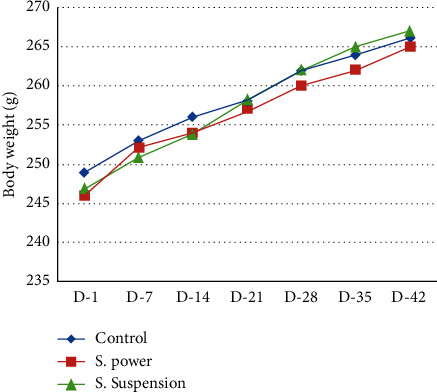
Mean body weights of rats during 42 days.

No significant changes in general behavior or other major physiological activities of rats were observed at any time point in this study. No significant changes were recorded in body weight and daily food intake in the treated rats as compared to the control. Both the control and treated rats appeared consistently healthy throughout the 42-day period of study. No death was also recorded in both the control and treated rats (Figure 1).

#### 3.2.2. Effect of Oral Administration of SP and SS on the Haematological and Biochemical Blood Parameters of the Rats

The haematological parameters (i.e., WBC, RBC, Hb, PCV, MCV, MCH, MCHC, RDW, MPV, and platelets) and the blood biochemical parameters (i.e., ALT, AST, ALP, ɤ-GT, urea, and creatinine) showed no significant changes (*p* > 0.05) in the treated rats compared to those in the the control rats, respectively. All levels were in consistent ranges until the termination of the study. The effects of SS and hot water extraction of SP after 42 days of oral administration are summarized in Tables 3–6.

#### 3.2.3. Effect of Oral Administration of SP and SS on the Wet Weights, Heights, Length, and Width of Rat Organs after 42-Day Treatment Period

The wet weights of rats' organs of both treated and control groups are presented in Table 5. The subchronic oral ingestion of SP and SS over 42 days caused no significant changes in the wet weights of the organs (i.e., heart, kidneys, and liver) in the treated groups and was compared with the control rats. The height, length, and width of rats' organs (i.e., heart, kidneys, and liver) of both treated and control groups after 42 days of oral ingestion are presented in Table 6. There were no significant changes in the outline measurements of rats' organs when compared to the control group.

#### 3.2.4. Histopathological Effect of Oral Administration of SP and SS on Selected Vital Organs after 42-Day Treatment Period

**Figure 2:**
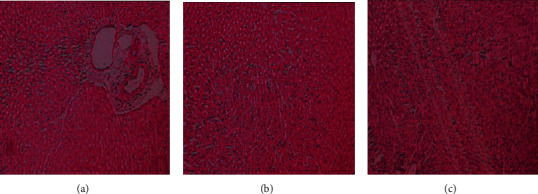
Photomicrograph of the liver tissue of the rats: (a) control; (b) SP; (c) SS. All the groups have a normal lobular architecture, normal sinusoids, and normal limiting plate hepatocytes. No vascular congestion, hepatocyte necrosis, or tissue degeneration were observed.

**Figure 3:**
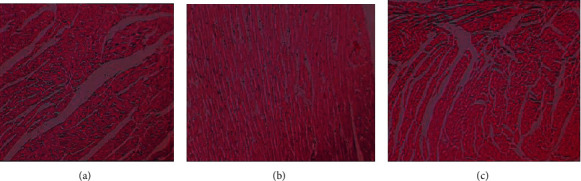
Photomicrograph of the heart tissue of the rats: (a) control; (b) SP; (c) SS. All the groups have a normal tissue structure.

**Figure 4:**
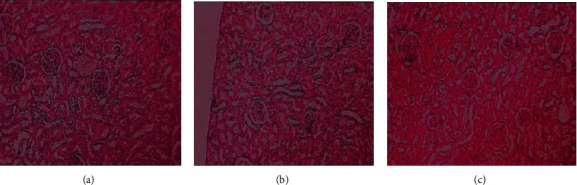
Photomicrograph of the kidney tissue of the rats: (a) control; (b) SP; (c) SS. All the groups have a normal tissue structure.

The liver, heart, and kidneys did not reveal any morphological changes on gross and histological examinations (Figures 2–4).

### 3.3. Clinical Study on Healthy Volunteers

#### 3.3.1. Effect of SS on Renal and Hepatic Functions

According to the findings of the healthy volunteers' study, there were no statistically significant (*p* > 0.05) differences in the serum parameters (ALT, AST, ALP, *γ*-GT, urea, creatinine, and Hb) on days 3 and 8 when compared with the baseline values (day 0) as shown in Table 7.

Healthy volunteers did not get any adverse effects, such as vomiting, headache, diarrhoea, or any other abnormal feeling during the treatment period and also during the follow-up period.

## 4. Discussion

In Ayurveda, the majority of natural drugs are of plant origin. Different plant parts such as roots, seeds, flowers, stem, bark, wood, leaves, and the plant as a whole are used as medicine. The medicinal property of the crude drug is due to the biologically active chemical constituents. The development of these chemical constituents is greatly influenced by different environmental and ecological factors. Ayurveda, allopathic medicine, and homeopathy are popular in society to achieve the same. Its widespread use is further substantiated by the affordability, knowledge of medicinal plants, and belief that they are harmless [12]. The increase in the number of users as opposed to the scarcity of scientific evidence on the safety of the medicinal plants has raised concerns regarding toxicity and detrimental effects of these remedies [13], and the same applies for novel preparation Sudarshana suspension.

There are many traditional herbals or polyherbal medicines that have not been verified by clinical trials, and hence, their efficacy and safety are still questioned by consumers. Drug safety is a very basic and fundamental concept in medical practice, and hence, the safety profile of SS and SP was investigated on male Wistar rats and healthy volunteers. The results obtained from the acute toxicity study showed that the SP and SS did not exert any possible toxicity in rats with single doses of drug administration (Tables 1 and 2). In the subchronic toxicity study, when the extract was administered daily to the animals for a period of 42 days, no mortality or morbidity was observed (Table 3) and no significant changes occurred in the haematological parameters (Table 4). The SS and SP did not cause any significant change in body weight when compared with the control group (Figure 1). The gross examination of internal organs revealed no detectable inflammation, while the weight of the animals treated with the SS and SP was not significantly different when compared to the control group (Table 5). According to reports, body and internal organ weights are considered sensitive indices of nontoxicity after 42 consecutive days of drug administration (Table 6). Serum concentrations of alanine amino transaminase (ALT) and aspartate amino transaminase (AST) are known to increase significantly in liver toxicity [14, 15]. Since, in this study, the enzymes showed no appreciable increase in the treated animals, it implied that the SS and SP have no hepatotoxic effect. This was confirmed by the histological study in which tissue morphology showed no changes (Figures 2–4).

The serum creatinine and urea levels too were not significantly altered compared to the control indicating no possible nephrotoxicity which was confirmed by the histopathological study as well. When haematological parameters were evaluated, mean corpuscular volume (MCV), mean corpuscular haemoglobin (MCH), and mean corpuscular haemoglobin concentration (MCHC) showed no significant variations following the treatment with SS. The importance of MCH, MCV, and MCHC in the diagnosis of anaemia in most animals has been highlighted. From this result, it has been shown that the SS and SP did not significantly alter the calculated RBC indices. The inflammatory process is characterized by the involvement of multiple inflammatory WBC [16]. In this study, it has not been observed that WBC has altered significantly while lymphocytes, the main effector cells of the immune system [17], showed no significant differences, thus suggesting that the SS and SP did not exert any toxic effect.

The findings from this part of the study provided sufficient data on the therapeutic safety of this polyherbal drug. It was observed that SS and SP do not exert any hepatotoxic effect that would cause the liver to compromise its function. Furthermore, the indices pertaining to renal function showed no abnormalities. The findings were further supported by the histological study where the hepatic, renal, and cardiac tissues showed no changes following SS and SP treatment. This paved the way for the SS to be tested in human individuals.

In new drug development, Phase I studies are the studies where a drug is initially given to human beings. These studies with more benign drugs often use healthy volunteers. Drugs are then initiated at very low doses and slowly escalated to show safety at a level where some biological activities take place. Later, when pharmacological and safety information is available, the drug is introduced to the patient population, again with an emphasis on safety [18]. Therefore, based on the above guidance, Phase I study of SS was carried out with healthy volunteers. According to the findings of the Phase I safety study, there were no statistically significant (*p* > 0.05) differences in the serum parameters (ALT, AST, ALP, *γ*-GT, urea, creatinine, and Hb) on days 3, 8 when compared with the baseline values (day 0) (Table 7).

Hence, *Sudarshana* suspension does not affect the above parameters when administered at 6 h intervals daily for 7 consecutive days. Therefore, it was revealed that it is well tolerated by the administered human therapeutic dose.

## 5. Conclusion

In conclusion, the hot water extract of SP and novel preparation SS given orally to male Wistar rats and healthy volunteers did not produce toxic effect at the therapeutic dose level when compared to the control. Therefore, SP and SS can be considered to be safe drugs for oral administration.

## Figures and Tables

**Table 1:** Effects of SS and hot water extraction of SP after 48 hours of oral administration.

Biochemical parameters	Groups		
	Control	SP	SS
Haemoglobin (Hb) (g/dl)	14.15 ± 0.11	13.92 ± 0.24	14.02 ± 0.19
AST/GOT (IU/L)	86.05 ± 1.9	83.44 ± 1.92	84.75 ± 1.53
ALT/TGP (U/L)	30.92 ± 0.80	32.22 ± 1.78	34.04 ± 1.30
Gamma GT (IU/L)	3.9 ± 0.71	3.91 ± 0.50	3.90 ± 0.59
Alkaline phosphatase (IU/L)	131.64 ± 2.03	132.05 ± 3.0	131.92 ± 2.15
Urea (mmol/L)	28.63 ± 1.24	30.92 ± 1.84	32.73 ± 1.38
Creatinine (mg/dl)	0.59 ± 0.02	0.62 ± 0.02	0.65 ± 0.02

Values are expressed as mean ± SEM; *n* = 35.

**Table 2:** Effects of SS and hot water extraction of SP after 14 days of oral administration.

Biochemical parameters	Groups		
	Control	SP	SS
Haemoglobin (Hb) (g/dl)	14.12 ± 0.08	14.01 ± 0.21	14.05 ± 0.23
AST/GOT (IU/L)	85.41 ± 1.83	83.0 ± 1.92	85.32 ± 1.38
ALT/TGP (U/L)	30.2 ± 0.86	32.41 ± 1.21	33.24 ± 1.24
Gamma GT (IU/L)	4.12 ± 0.53	4.05 ± 0.36	4.05 ± 0.43
Alkaline phosphatase (IU/L)	133.64 ± 2.08	134.58 ± 2.75	134.47 ± 1.32
Urea (mg/dl)	28.59 ± 2.01	28.87 ± 1.43	31.23 ± 1.10
Creatinine (mg/dl)	0.60 ± 0.03	0.64 ± 0.01	0.63 ± 0.02

Values are expressed as mean ± SEM; *n* = 35.

**Table 3:** Effects of oral administration of SP and SS on the biochemical parameters of rats' blood after 42-day period of oral administration.

Biochemical parameters	Groups		
	Control	S. powder	S. suspension
AST/GOT (IU/L)	85.97 ± 1.81	84.07 ± 1.86	85.17 ± 1.43
ALT/TGP (IU/L)	31.5 ± 0.62	32.92 ± 1.31	34.01 ± 1.76
Gamma GT (IU/L)	4.21 ± 0.61	4.37 ± 0.43	4.07 ± 0.43
Alkaline Phosphatase(IU/L)	135.81 ± 2.91	136 ± 3.91	133.51 ± 2.25
Urea UV (mg/dl)	32.57 ± 1.49	32.11 ± 1.77	33.25 ± 1.02
Creatinine (mg/dl)	0.62 ± 0.02	0.67 ± 0.01	0.67 ± 0.02

Values are expressed as mean ± SEM; *n* = 6.

**Table 4:** Effects of oral administration of SP and SS on the haematological parameters of rats' blood after 42-day period of oral administration.

Blood parameters	Groups		
	Control	S. powder	S. suspension
White blood cells (WBC) (10^3^/*μ*l)	8.18 ± 0.17	7.9 ± 0.13	8.02 ± 0.15
Neutrophil (%)	33.18 ± 3.2	28.65 ± 2.80	32.21 ± 2.82
Lymphocytes (%)	55.44 ± 1.84	59.54 ± 2.60	56.88 ± 2.58
Monocyte (%)	4.35 ± 1.23	5.92 ± 1.40	5.35 ± 1.0
Eosinophil (%)	2.54 ± 0.55	1.78 ± 0.18	2.07 ± 0.40
Basophil (%)	2.6 ± 0.70	3.8 ± 0.67	3.5 ± 0.53
Red blood cells (RBC) (10^6^/*μ*L)	8.29 ± 0.35	7.8 ± 0.19	7.9 ± 0.19
Haemoglobin (Hb)	14.18 ± 0.39	14.3 ± 0.20	14.18 ± 0.27
Packed cell volume (PCV) (%)	48.94 ± 1.85	48.65 ± 1.26	48.41 ± 1.93
Mean corpuscular volume (MCV) (fL)	53.61 ± 2.15	53.97 ± 1.78	56.37 ± 1.51
Mean corpuscular haemoglobin (MCH) (pg)	24.05 ± 1.27	23.37 ± 0.86	24.05 ± 0.97
Mean corpuscular haemoglobin conc. (MCHC) (pg)	27.34 ± 1.08	26.8 ± 0.91	25.87 ± 1.28
RDW (%)	14.81 ± 0.11	15.0 ± 0.19	14.52 ± 0.08
Platelets (10^3^/*μ*L)	636.42 ± 2.73	631.71 ± 2.02	635.42 ± 2.28
MPV (fL)	3.25 ± 0.42	2.61 ± 0.16	2.7 ± 0.11

Values are expressed as mean ± SEM; *n* = 6.

**Table 5:** Wet weight of the heart, kidneys, and liver of rats chronically treated with SP and SS.

Groups	Organ weight (g)			
	Liver	Heart	R. kidney	L. kidney
Control	13.80 ± 0.40	1.12 ± 0.04	1.20 ± 0.02	1.13 ± 0.02
S. powder	13.66 ± 0.36	1.18 ± 0.04	1.23 ± 0.03	1.16 ± 0.04
S. suspension	14.17 ± 0.32	1.15 ± 0.03	1.23 ± 0.02	1.15 ± 0.01

Values are expressed as mean ± SEM; *n* = 6.

**Table 6:** Height, length, and width of rats' organs chronically treated with SP and SS.

Groups	Organ height × length × width (cm)			
	Liver	Heart	R. kidney	L. kidney
Control	2.18 ± 0.09	0.94 ± 0.02	0.88 ± 0.06	0.8 ± 0.04
	5.57 ± 0.32	1.85 ± 0.05	2.11 ± 0.10	1.92 ± 0.07
	4.6 ± 0.18	1.31 ± 0.03	1.24 ± 0.14	1.24 ± 0.09
S. powder	2.18 ± 0.07	0.8 ± 0.02	0.71 ± 0.02	0.67 ± 0.01
	5.57 ± 0.07	1.8 ± 0.03	1.88 ± 0.02	1.71 ± 0.04
	4.42 ± 0.02	1.28 ± 0.02	1.47 ± 0.03	1.38 ± 0.02
S. suspension	2.1 ± 0.06	0.8 ± 0.03±	0.7 ± 0.02	0.67 ± 0.01
	5.11 ± 0.12	1.84 ± 0.02	1.94 ± 0.02	1.81 ± 0.02
	4.31 ± 0.06	1.27 ± 0.01	1.37 ± 0.01	1.32 ± 0.02

Values are expressed as mean ± SEM; *n* = 6.

**Table 7:** Effect on renal and hepatic functions following 7 days of oral administration of SS.

Biochemical parameters	Testing days and *p* values				
	Day 0	Day 3	*p* value	Day 8	*p* value
Haemoglobin (Hb) (g/dl)	12.71 ± 0.24	12.70 ± 0.24	0.28	12.74 ± 0.23	0.19
AST/GOT (IU/L)	28.86 ± 0.68	28.57 ± 0.73	0.12	28.89 ± 0.69	0.46
ALT/TGP (U/L)	23.12 ± 1.44	22.76 ± 1.42	0.16	23.20 ± 1.43	0.4
Gamma GT (IU/L)	11.68 ± 0.85	11.54 ± 0.79	0.35	11.29 ± 0.81	0.7
Alkaline phosphatase (IU/L)	210.05 ± 8.44	209.13 ± 8.20	0.12	209.10 ± 8.15	0.11
Urea (mmol/L)	2.83 ± 0.13	2.80 ± 0.13	0.19	2.84 ± 0.12	0.4
Creatinine (mg/dl)	0.78 ± 0.01	0.78 ± 0.01	0.49	0.79 ± 0.01	0.25

All the values are expressed as mean ± SEM; *n* = 35. *p* > 0.05, not significant.

## Data Availability

The data used to support the findings of this study are available from the corresponding author upon request.
